# TELE-PSYCHOTHERAPY OF ANXIETY AND DEPRESSION DISORDERS

**DOI:** 10.1192/j.eurpsy.2023.1431

**Published:** 2023-07-19

**Authors:** I. S. Lancia, G. M. Festa, A. Attouchi, M. Civino

**Affiliations:** 1 Interdisciplinary Institute of Higher Clinical Education (IAFeC), Naples; 2 Interdisciplinary Institute of Higher Clinical Education (IAFeC); 3 Pontifical Faculty of Educational Sciences «AUXILIUM», Rome, Italy

## Abstract

**Introduction:**

The process of integrating technology into mental health pathways represents a social transformation that we are gradually getting used to. But does it represent a valid alternative to face-to-face care processes? In this paper we will consider telesychology as a tool for treating anxiety and depression and its validity. Anxiety and depression are harmful to individuals, suffering from these disorders, their caregivers, and the economy. Remote delivery of psychotherapy has been established as a viable alternative to traditional in-person psychotherapy for treating anxiety and depression. However, literature comparing and evaluating the variety of remote delivery modalities of psychotherapy has not yet been integrated.

**Objectives:**

This review examines the efficiency – to - practice and the limits of e-therapy and its mediums: telephone, video, and online-administered psychotherapy, for treatment of anxiety and depression.

**Methods:**

A comprehensive literature search, conducted using PubMed and PsycINFO included systematic reviews, randomized controlled trials, and cost-analysis studies focused on a remote delivery method of e-psychotherapy for anxiety and depression

**Results:**

Overall, interventions delivered through telephone, video, and online modalities, have generally demonstrated good efficiency in treating anxiety and depression; also comorbid with other disorders. The literature also suggested that telehealth psychotherapy is accessible, convenient, and cost-effective.

In evaluating the reviews on the databases, it also emerged that among the many psychological therapies for anxiety disorders, delivered digitally (CBT, Attention bias modification, Exposure therapy, Applied relaxation, Bibliotherapy, Psychodynamic therapy, Mindfulness, Behavioral stress management, Counseling), the best digital therapy is internet-based cognitive behavioral therapy (iCBT), in particular for Social Anxiety Disorder (SAD).

Despite this, overall, the efficiency and practical benefits of remote psychotherapy interventions in treating anxiety and depression across a diverse range of patient groups suggested that it is an appropriate alternative for those who cannot access in-person psychotherapy.

**Conclusions:**

Further research evaluating the efficiency and practical benefits of e-psychotherapy for anxiety and depression is much needed for patients with limited access to in-person psychological care. Moreover, it remains to evaluate the maintenance of therapeutic gains after the end of the treatments.

**Disclosure of Interest:**

None Declared

